# Pricing Externalities to Balance Public Risks and Benefits of Research

**DOI:** 10.1089/hs.2016.0118

**Published:** 2017-08-01

**Authors:** Sebastian Farquhar, Owen Cotton-Barratt, Andrew Snyder-Beattie

## Abstract

How should scientific funders evaluate research with public health risks? Some risky work is valuable, but accepting too much risk may be ethically neglectful. Recent controversy over H5N1 influenza experiments has highlighted the difficulty of this problem. Advocates of the research claim the work is needed to understand pandemics, while opponents claim that accidents or misuse could release the very pandemic the work is meant to prevent. In an attempt to resolve the debate, the US government sponsored an independent evaluation that successfully produced a quantitative estimate of the risks involved, but only a qualitative estimate of the benefits. Given the difficulties of this “apples-to-oranges” risk-benefit analysis, what is the best way forward? Here we outline a general approach for balancing risks and benefits of research with public risks. Instead of directly comparing risks and benefits, our approach requires only an estimate of risk, which is then translated into a financial price. This estimate can be obtained either through a centrally commissioned risk assessment or by mandating liability insurance, which allows private markets to estimate the financial burden of risky research. The resulting price can then be included in the cost of the research, enabling funders to evaluate grants as usual—comparing the scientific merits of a project against its full cost to society. This approach has the advantage of aligning incentives by assigning costs to those responsible for risks. It also keeps scientific funding decisions in the hands of scientists, while involving the public on questions of values and risk experts on risk evaluation.

## Abstract 2

How should scientific funders evaluate research with public health risks? Some risky work is valuable, but accepting too much risk may be ethically neglectful. Recent controversy over H5N1 influenza experiments has highlighted the difficulty of this problem. The authors outline a general approach for balancing risks and benefits of research with public risks.

In 2012, multiple research groups conducted experiments that produced strains of H5N1 avian influenza that were airborne transmissible between mammals.^1,2^ Given that this disease is often fatal in humans, these particular “gain-of-function” experiments sparked wide controversy^*^. Advocates of the research argued that the experiments were necessary to improve our understanding of the virus, thus enabling better disease surveillance and vaccine production. Opponents of the research argued that a laboratory accident could trigger a global pandemic, or that the research could be used by malicious actors.^3^ The debate culminated in a moratorium on US public funding of research “that may be reasonably anticipated to confer attributes to influenza, MERS, or SARS viruses such that the virus would have enhanced pathogenicity and/or transmissibility in mammals via the respiratory route.”^4^ Meanwhile, the US government also commissioned an independent study from Gryphon Scientific to weigh the benefits of the research against the possible biosafety and biosecurity risks.^5^ While their research succeeded in producing a quantitative estimate of biosafety risk, it explained the benefits of the research only in qualitative terms.

This controversy highlights the difficulty of evaluating research that has the potential to entail significant public risk. Research that is particularly likely to lead to both positive and negative impacts is especially hard to evaluate.^6^ These negative consequences might be either the risk of an accident linked directly to the research itself, or risks of deliberate misuse of the resulting technologies. Avoiding research that has any theoretical risk at all would mean missing out on crucial opportunities. But research communities that underestimate the potential for a significant accident, or assume that people will use information gained through research only for good, may be neglecting their duty to society.^7^

The H5N1 flu experiments mark a particularly interesting case study that we use to highlight some problems in evaluating the risks and benefits of dual-use research.^†^ In this article, we discuss some of the current difficulties of existing approaches to evaluating risky research, and we propose a general framework that avoids many of the current problems. Within this framework, we outline 2 possible approaches to implementing the framework: a market-led liability insurance approach, and a state-led approach. We conclude by comparing the approaches with each other and with current policy.

## Difficulties of Risk-Benefit Analysis for Scientific Research

Risk-benefit analysis, the approach used in the Gryphon Scientific report, is an analytical tool that has been successfully applied to a wide range of domains.^8^ Analysts outline a range of scenarios with positive and negative impacts, estimate the probabilities of those scenarios and the net size of the positive impact, and compute the expected value of the proposal. When attempting to do a risk-benefit analysis of research, determining the scenarios and the probabilities of each scenario can be difficult. But as it turns out, estimating biosafety risks is far easier than estimating the benefits of research. The reason for this is that one can outline the potential scenarios for biosafety risks fairly well. In order for a laboratory accident to cause a global pandemic, a set of events must occur. The probability of each of these steps can then be estimated using historical data on laboratory accidents and models of disease spread. These estimates are highly uncertain, because of limitations in available data. The data describing exactly what activities labs engage in and their historical failure rates are incomplete, though some records are kept.

Moreover, assumptions are required to extrapolate past records into the future. For example, lab safety standards and equipment have improved over the past several decades, but at the same time new labs are being built under the jurisdiction of regulators with less of a track record establishing their biosafety capabilities. Risk estimators must also make some estimate of the probability distribution of key parameters that cannot be empirically tested. For example, what is the probability that a reassortant virus will be sufficiently transmissible to spread to a wide population? Nevertheless, quantitative estimates are possible, though they lead to disagreement.^9-11^ Many risks that are in principle very difficult to estimate for similar reasons are nevertheless quantitatively priced by insurers today—such as terrorism,^12^ cyberattack,^13^ and harm to patients in clinical trials.^14^

Conversely, estimating the benefits of research is extremely difficult. Unlike estimating the probability of a laboratory accident scenario, evaluating the benefits of research involves uncertainty over what the possible scenarios even are. Typically, researchers have little information about the potential results of research—which is one of the motivations to do research in the first place. Even the most experienced researchers find it difficult to estimate the ex-ante value of new research projects. The work recreating the genome of the influenza virus responsible for the 1918 pandemic^15^ received some criticism for vaguely promising unspecified future benefits^16^ but, after some time, has plausibly created tangible benefits including, perhaps, aiding in the management of the 2009 H1N1 pandemic, according to the authors of the original paper.^17^ In their review, Gryphon Scientific did not even attempt to assess the benefits of research quantitatively, and it is this part of the risk-benefit calculation that typically poses the greatest difficulty.^5,18^

Evaluating the risks from malicious activity is more difficult than estimating the risk of accidents, but easier than estimating the benefits of research. Ultimately, the Gryphon Scientific report included a quantitative assessment of some biosecurity risks (though not the risks posed by the information discovered by the research). Unlike the accident scenarios, uncertainties around both the intentions and capabilities of malicious actors mean a wider range of scenarios is possible. But despite the difficulties in quantifying such risk, many insurance companies still offer terrorism insurance, and models do exist for putting rough numbers on such risks.^12^

When science funders evaluate dual-use research, they are faced with risks and benefits that are uncertain in different ways. Most of the risks are quantitatively estimable, but the parameters are hard to pin down. It is our view that, even when highly uncertain, explicit quantitative risk estimates are preferable to implicit ones, because they allow transparent discussion about disagreements and mitigate human biases in judgments around small probabilities.^19^ The benefits, however, are so uncertain that even specifying the scenarios is impossible. Fortunately, existing grant-making approaches are already designed to compare projects with nebulous benefits. So, as long as the quantified risks are accounted for in the cost of a project, these existing systems are well suited for evaluating this research.

## Existing Approaches and the Scientific Grant-Making Process

Grant makers already assess the uncertain benefits of research against their costs and the opportunity costs of unfunded research. Although it is hard to judge quantitatively, reviewers assess the potential for scientific excellence in different proposals.

However, the costs that reviewers consider are primarily direct financial costs, rather than externalities such as the risks borne by the public from dual-use research. In principle, research funders prefer to fund portfolios of research with maximal benefits of research for society (including positive externalities) and minimal costs (including negative externalities). The concept of scientific merit already includes some sense of positive externalities, and the UK, at least, also considers the social impact of research and its effect on the research ecosystem.^20^ Conceptual tools and intuitions on risk externalities are less well developed.

Reviewers often implicitly evaluate dual-use risk as a matter of personal or professional ethics^21^ or for the sake of reputation of the field. However, for most research, the dual-use stakes are low. In these situations, researchers have no need to also become experts in risk assessment and management. Research institutions rely instead on safety regulations for particular categories of risk in order to make sure research processes are safe.^22^ These tools work very well when most of the risk is local, for accidental releases at the community level, or for health risks to lab researchers themselves. Historically, this is where most of the risk in biological research has lain and is therefore an entirely appropriate default.

However, in the case of risks that carry a small chance of large catastrophe (eg, research that creates potential pandemic pathogens), these regulations are not enough. Some funders, such as the US government, provide additional decision-process guidelines for funding research involving specific pathogens, like H5N1,^23^ as well as institutional guidelines for best practice for dual-use research.^24^ Others, such as the UK's Biotechnology and Biological Sciences Research Council, the Medical Research Council, and the Wellcome Trust, require grant applicants to consider near- and medium-term risks of misuse but rely heavily on self-governance and a responsible research culture.^25^ These frameworks put a high burden on those pursuing dual-use research to become risk assessors. Moreover, public funding guidelines only affect government funding and cooperative private funders. Private dual-use research is less constrained.^26^

Some additional judgment can in principle be applied at the stage of publication,^27^ but a 2012 survey of 127 life sciences journals found that none reported refusing a submission on biosecurity grounds.^28^ Export controls may also be able to restrict some kinds of publication^29^ in some jurisdictions. It may be that under the Australia Group's “no undercut” principle, in its guidelines for transfers of sensitive chemical or biological weapons, if any participant nation were to block publication using export controls then all would be obliged to (though the mechanisms for applying the guidelines to research results is unclear).^30^

## Pricing and Using Externalities in Grant Making

### Absolute Risk as a Cost of a Grant

Hard-and-fast rules about what research techniques are too risky, without considering the benefits, may not be responsive enough, because more important research merits more risk.^31^

Our principal proposal is that one could assess and price absolute expected risk and explicitly include this cost in grant proposals. This helps resolve the fact that while risks can be comparatively easy to assess, the benefits of research are extremely challenging to quantify. Our proposal allows grant reviewers to use existing frameworks to evaluate grant proposals but weigh potential benefits against a fairer reflection of their social costs. It also follows calls to strengthen the evaluation of risks at pre-funding stages of the research process.^32^

For now, we set aside the issues of where an independent estimate of the cost of externalities comes from and where the money to cover this explicit cost is paid. The 2 main approaches to setting estimates, which we consider in detail in the next section, are:

1. To establish clear liability in case of catastrophe and require grant holders to purchase liability insurance as part of the grant. The strength of this is that it is a market-led approach, with insurers incentivized to price externalities correctly.
2. To centrally commission absolute risk assessments to price the externalities, and to require a payment to a state or non-state body to cover the expected cost. The strength of this approach is that it works even if there may be no clear liability after the fact, so it could address biosecurity as well as biosafety risks.

### Benefits of the Approaches

Both approaches have 4 main advantages. First, they give decision makers greater incentive to use an accurately priced risk when deciding what research to fund. Currently, the costs of accidental or deliberate harm from gain-of-function research of concern are borne by members of the public in any country as well as lab workers. For example, after a foot-and-mouth disease outbreak in Pirbright in 2007, caused by poorly maintained drainage,^33^ prosecutors were unable to seek damages because there was no demonstrable negligence of a specific party.^34^ Instead, governments bore the financial cost of compulsory slaughter, farmers bore the costs of damaged and restricted business, and consumers experienced higher food prices. Where the risks of an activity are not borne by those making decisions about the activity, economic theory predicts that more risk will be taken than is socially optimal.^35^ So long as the risks are priced correctly and the transaction costs are sufficiently low, research institutions that pay upfront for the risks of research are more likely to engage in socially optimal risk-taking. If implemented successfully, this would result in both better grant choices and better use of risk reducing techniques and equipment.

Second, these approaches keep decisions about what research gets funded in the hands of researchers. One of the largest difficulties in assessing the social value of research is assessing the intellectual benefits of research. Researchers themselves are best placed to evaluate the potential for success and the intellectual merit of research because they have the deepest knowledge of the area.

Third, these approaches incorporate democratic or public input on the significance of risks. Although researchers have expertise in the potential for intellectual success from a grant proposal, they are not necessarily able to speak for the values of society. Researchers have an obligation to incorporate public input on value systems and consider the justice implications of their work.^31^ Our proposal creates ways for the public to express their values without creating rules allowing or disallowing certain research techniques or areas of inquiry. In the case of an insurance mechanism, the legislative branch effectively determines the social cost borne were the risk to materialize, via the judiciary. In the case of a central body setting the costs, this body would ultimately be accountable to the electorate.

Fourth, these approaches ensure appropriate risk assessment and governance expertise is brought to bear. Researchers submitting grant proposals are often asked to consider potential dual-use and accident risk. Grant makers do so similarly. However, neither group tends to include specialists in risk assessment or biosecurity.^26^ If absolute risk is priced separately, the price can be set by expert risk assessors.

Throughout, our guiding principle is to ensure the right incentives are in place and that components of the funding decision are broken up such that those best placed to assess them do so.

### Disadvantages of the Approaches

Although we believe these approaches might improve the capacity of the research funding system to manage catastrophic risk, they introduce some new concerns.

First, research institutions currently receive a subsidy in the form of an implied guarantee covering the costs of imposed risks. If they were asked to pay for this guarantee, it would represent an additional unfunded expense. Since the aim of this proposal is to distribute research effort within, say, the life sciences rather than to change the absolute amount of life science work being done, one might increase funding for life sciences research to compensate. This would appear to increase explicit expenditure in government research budgets, but in fact moves an implicit, random, and large subsidy from the budgets of disaster response agencies, who do not make decisions that determine the risk exposure, to an explicit, predictable, and moderate payment by those who do make such decisions. For example, governments and consumers bear a large part of the cost of escapes of animal pathogens from research labs^36^ but have less influence over risk exposure than research funding bodies.

Second, penalizing laboratories for reporting accidents and near misses in a timely way harms biosafety and biosecurity in the long run. Increasing reporting would make it easier to use lessons from mistakes to improve lab design and improve accident response. Mechanisms for pricing risk will work best if they avoid creating perverse incentives around reporting. Options for facilitating this include simply not using the information from recent accidents in the case of central analysis, or forbidding a premium hike in the case of liability insurance requirements.

Third, these approaches increase bureaucratic overhead. There are several things that might be done to reduce this overhead. One could apply our proposal only to areas with particularly high risk of low-probability but high-impact events. For example, it might be applied only to the gain-of-function research that has been covered by the recent US moratorium. Or rather than carrying out a separate risk analysis for each proposal, one could establish broad categories of similar work and set costs for that category as a whole. For some categories, it might be possible to get involvement from intelligence communities to incorporate information not normally available. In the future, scope could be expanded by addressing new areas or by making the cost estimates more granular.

### Making Decisions Under Uncertainty

There are things these approaches would not fix. Risk in many cases of dual-use research is very uncertain. No risk management or risk governance process can solve this entirely. It is hoped that in the case of market-led approaches, the profit motive of companies providing insurance as well as an awareness of “winners' curse” dynamics will incentivize appropriate pricing. In the case of a state-led approach, it is hoped that the system will increase the capacity for risk-assessment by developing a core of expert assessors. Political pressures or “short-termism” might cause prices to be inaccurate. However, this is also a feature of existing approaches to dual-use risk management.

## Approaches to Risk Pricing

### Mandatory Liability Insurance

Laboratories conducting experiments of appropriate kinds could be mandated to purchase insurance against liability claims arising from damaging accidents from their research. Insurance companies would then pay out to injured parties in the public in case of an accident. Some proposals in this direction have already been discussed in the UK.^27^ In some cases, appropriate use of liability for damages is more effective than regulation in encouraging risk-reducing behavior.^37^ In most jurisdictions, negligence leading to catastrophic damage would already establish liability. Sometimes, the complexity of the research process makes it hard to establish negligence of a specific party.^34^ Governments might therefore need to legislate to establish clear duty of liability in any damages caused by accident, even if there was no negligence. Liability would give universities strong incentives to minimize the risk. Framers of this legislation would need to be quite specific about which sorts of liability were included (eg, health damage) and which were not (eg, adversely affected tourism, perhaps, or terrorism). Such an approach might be similar to some aspects of the 1957 Price-Anderson Act, which placed a strict liability on operators of potentially risky private nuclear facilities and a mandate to purchase the highest commercially available amount of insurance coverage.^38^ A similar approach has been tried and advocated for in environmental risk, though it proved hard to maintain a healthy insurance market.^39,40^

Requiring insurance is important because some risk takers will not be able to compensate victims for catastrophic harms. Many universities currently self-insure against the damage of accidents in their research. Where there is a small chance of catastrophic damage, they do not have enough assets to cover the damages—they are “judgment-proof.” Insurance providers, with much greater financial liquidity, are able to bear the full costs even when payouts are very large, which incentivizes them to set premiums appropriately.^41^ For the most catastrophic forms of damage, such as a global pandemic, costs would be too big even for a reinsurer to bear. As a result, an insurance approach could involve an explicitly negotiated liability cap, in which case regulatory bodies tasked with managing the most extreme forms of risk are essential.

Various amendments to the Price-Anderson Act demonstrate the problems facing liability limits. Indemnifying those creating the risk from all costs above the privately insurable can cause excess risk taking.^42^ The most recent version of Price-Anderson requires regulated nuclear energy providers to pool to jointly cover more than $10 billion of costs beyond that covered for each individual party by insurers. While this protects the public by identifying a source of funds beforehand, the fact that the individual contributions do not need to be set aside in advance means that the entire industry carries a large, uninsured, systemic risk. A modern system based on catastrophe bonds might offer a safer way to increase the feasible levels of financing.

### Advantages of the Liability Approach

There are 2 main advantages to this market-led approach. First, it is a relatively light intervention, requiring little ongoing work from the state (other than maintaining a healthy insurance market). Second, the insurers would have a profit incentive to accurately estimate the risk, reducing possible politicization of the risk assessment process. In a sufficiently liquid market, competitive pressures should bring premiums down. But insurers are aware of a “winners' curse” in the insurance market and would avoid setting premiums too low to cover the long-term risk. If insurers adjust their premiums when safer systems and equipment are adopted, scientists and engineers would be incentivized to devise effective safety protocols to reduce their institutions' insurance costs. This would create a financial driver for applied biosafety and biosecurity research. Imposing liability has improved outcomes in other domains such as occupational safety, medicine, and general risk management in nonprofits and government agencies.^43^

### Possible Issues with the Liability Approach

Insurers must be willing to insure against biosafety and biosecurity risk. While insurers have shown some interest,^44^ there is no functioning market for the same 2 main reasons insurers were unwilling to take on nuclear risk before the 1957 Price-Anderson Act:^38^

First, the potential risks are simply too large. A catastrophic global pandemic could kill hundreds of millions of people, and even the largest reinsurers would be unable to absorb this cost without bankrupting themselves (costs above this level will be implicitly backed by the state or the public in any case). It is better to be explicit and cap liability at a specific industry-wide figure. If the cap were sufficiently large, the effect should be more appropriate risk aversion, even if the tail risk for the insurer were not fully internalized.

Second, the risks are hard to model and the market would be quite small. Developing models to estimate the risk could be more costly than the expected profit from being in the market. However, insurers do already have models for hard-to-anticipate risks such as terrorism and global pandemics. If need be, the development of appropriate models to facilitate this insurance could be explicitly subsidized.

Whether or not insurers are willing to take on the risk, there are challenges to international adoption. Liability laws differ across jurisdictions, which would particularly affect international collaborations. One effective work-around might be to form an agreement among top-tier research journals to require proof of suitable liability insurance for some types of papers.^‡^

Liability insurance can also increase moral hazard, by making actors less responsible for the consequences of their actions. This effect could be reduced if the excess on the insurance were large enough that research institutions still had a direct incentive to avoid risks.

Finally, using a liability approach to capture biosecurity risks would be difficult. If a terrorist group used information they gleaned from a paper published by a research group, establishing liability would require lengthy legal dispute. Moreover, done carelessly, the uncertainty additional but unclear liability could create might have an inappropriate chilling effect on research.

### Centrally Commissioned Risk Assessments

The second approach is to centralize risk assessments. When an area of potential concern is identified, a body commissioned by the state would perform an analysis of the risks involved. This might be similar to the recent Gryphon Scientific analysis, except that it would not attempt to analyze the benefits. This absolute risk analysis would present its outcomes in monetary terms, using value of statistical life figures to convert into a cost.

In order to do work of a priced type, laboratories would have to pay the appropriate charge to a central authority. This charge could cover part of the risk of biosafety and biosecurity preparation as well as the cost of risk assessment.

### Advantages of the Centrally Commissioned Risk Assessment

Compared to the market-led approach, centralizing the risk assessments has 2 main benefits. First, it can be done by fiat without needing to persuade insurers to enter and stay in the market. Second, it works well even in cases where there will be no clear causal chain establishing liability, such as for biosecurity risks (including risks from the misuse of information produced from such research). Funds are collected based on the central assessment of the size of the risk posed by the research, with no need to work out after the fact which specific group is liable. The Gryphon Scientific report concluded that the biosecurity risks looked at least as large as the biosafety risks, so this is a significant benefit in the case of gain-of-function research of concern.^5^ Third, at least in principle, avoiding the need for courts to establish liability might speed up payments.

### Possible Issues with the Central Approach

Patchy implementation only in certain jurisdictions might just move research from nations with good risk management processes to those without. In practice, however, many researchers are likely to want to remain at top labs, limiting the risk of research simply moving to jurisdictions that do not assess the externalities of research. There may be a need for special arrangements for research done in partnership with labs outside the collecting jurisdiction, such as applying a requirement for proof of insurance at the stage of publishing.

The risk assessment itself would be difficult. The centrally commissioned assessment would be more likely than an insurance-led one to need to assess the magnitude of biosecurity risks, which is particularly hard to model. However, stakeholders might have incentives to put pressure on regulators to make certain decisions. This is more likely to be successful because of the amount of judgment involved in the calculations of risk. Unlike the insurance-led solution, there would be no countervailing profit motive.

## Comparisons and Discussion

The 2 approaches discussed would work by aligning the incentives for scientists and for funding bodies more closely with those of society as a whole. This approach keeps assessment of the benefits of scientific research purely in the hands of scientists, while bringing the right skills to bear at the right points and managing risk proportionately. We have explored 2 different ways to achieve this. Each has its advantages and disadvantages.

The liability approach is more market based. As a result, the risk assessors have a financial incentive to accurately estimate risk, and political pressures are diminished. It might also be easier to use as a template internationally. Since the risks are global and the potentially risky research is not being pursued in just 1 country, being able to build global solutions is extremely valuable.

The main benefit of the centrally commissioned analysis is it could bypass lack of clarity about who is liable for eventual damages in cases of biosecurity.

These approaches have clear application for gain-of-function research of concern in the life sciences. However, similar approaches may work in a broader range of fields. The insurance mechanism is best suited for dual-use work where risk is predominantly through accident rather than malicious intent. In all cases, pricing the absolute size of the risk and leaving researchers in charge of decisions about how to spend their budgets might offer a more responsive and proportionate approach to the challenges of funding research with potential public risks than existing systems.

## Notes

^*^ Gain-of-function experiments encompass a wide range of research, and only a small subset of such experiments were subject to this controversy. In this article, we focus on gain-of-function research of concern, by which we mean research of the sort affected by the funding moratorium, as a case study of the more general problem of making research funding decisions for dual-use research of concern.

^†^ In the context of atomic technologies, discussions of dual-use technology focused on the ability to rapidly convert civilian technologies for military purposes,^8^ and this trend continued for much of the 20th century.^9^ Recently, discussions of dual-use research have broadened to include nonmilitary actors and the risk of accidents related to research.^5,10^ It is this last, broadest sense of dual-use research that we focus on in this article, as it captures the full spectrum of risks resulting from research activities. We acknowledge but do not address the fact that the definition of “dual-use” remains unsettled,^11^ and that one might want to consider dual-use research, technology built on that research, and artefacts implementing that technology separately.^9^

^‡^ We are grateful to Piers Millett for this suggestion.

## References

[1] Herfst S, Schrauwen EJ, Linster M, et al. Airborne transmission of influenza A/H5N1 virus between ferrets. Science 2012;336(6088):1534-1541 22723413 10.1126/science.1213362 PMC4810786

[2] Imai M, Watanabe T, Hatta M, et al. Experimental adaptation of an influenza H5 HA confers respiratory droplet transmission to a reassortant H5 HA/H1N1 virus in ferrets. Nature 2012;486(7403):420-428 22722205 10.1038/nature10831 PMC3388103

[3] Duprex WP, Fouchier RA, Imperiale MJ, Lipsitch M, Relman DA Gain-of-function experiments: time for a real debate. Nat Rev Microbiol 2014;13(1):58-64 25482289 10.1038/nrmicro3405 PMC7097416

[4] U.S. government gain-of-function deliberative process and research funding pause on selected gain-of-function research involving influenza, MERS, and SARS viruses. 2014 https://www.phe.gov/s3/dualuse/Documents/gain-of-function.pdf Accessed 71, 2017

[5] Risk and Benefit Analysis of Gain of Function Research. Takoma Park MD: Gryphon Scientific; 2016 http://www.gryphonscientific.com/wp-content/uploads/2016/04/Risk-and-Benefit-Analysis-of-Gain-of-Function-Research-Final-Report.pdf Accessed 71, 2017

[6] National Research Council; Policy and Global Affairs; Development, Security, and Cooperation; Committee on Research Standards and Practices to Prevent the Destructive Application of Biotechnology. Biotechnology Research in an Age of Terrorism: Confronting the Dual-Use Dilemma. Washington, DC: National Academies Press; 2004

[7] Kuhlau F, Eriksson S, Evers K, Höglund AT Taking due care: moral obligations in dual use research. Bioethics 2008;22(9):477-487 18959730 10.1111/j.1467-8519.2008.00695.x

[8] Wilson R, Crouch EAC Risk/Benefit Analysis. 2d ed. Cambridge, MA: Harvard University Press; 2001

[9] Casadevall A, Imperiale MJ Risks and benefits of gain-of-function experiments with pathogens of pandemic potential, such as influenza virus: a call for a science-based discussion. MBio 2014;5(4):3-7 10.1128/mBio.01730-14 PMC4128368 25085113

[10] Lipsitch M, Inglesby T V Moratorium on research intended to create novel potential pandemic pathogens. MBio 2014;5(6):1-7 10.1128/mBio.02366-14 PMC4271556 25505122

[11] Fouchier RA Studies on influenza virus transmission between ferrets: the public health risks revisited. MBio 2015;6(1):14-17 10.1128/mBio.02560-14 PMC4323420 25616377

[12] Woo G Quantitative terrorism risk assessment. J Risk Financ 2002;4(1):7-14

[13] Garrie D Cyber-security insurance: navigating the landscape of a growing field. John Marshall J Inf Technol Priv Law 2014;31(3):379-391

[14] Sagitova BK Watching clinical trials liability. Allianz Global Corporate & Specialty. 2009 http://www.agcs.allianz.com/insights/expert-risk-articles/watching-clinical-trials-liability/ Accessed 71, 2017

[15] Taubenberger J, Reid A, Lourens R, Wang R, Jin G, Fanning T Characterization of the 1918 influenza virus polymerase genes. Nature 2005;437(7060):889-893 16208372 10.1038/nature04230

[16] van Aken J When risk outweighs benefit. EMBO Rep 2006;7(S):10-13 16819441 10.1038/sj.embor.7400726 PMC1490308

[17] Taubenberger JK, Baltimore D, Doherty PC, et al. Reconstruction of the 1918 influenza virus: unexpected rewards from the past. MBio 2012;3(5):1-5 10.1128/mBio.00201-12 PMC3448162 22967978

[18] Risk and Benefit Analysis of Gain of Function Research. Draft Final Report. Takoma Park, MD: Gryphon Scientific; 12 2015 http://www.gryphonscientific.com/wp-content/uploads/2015/12/Final-Gain-of-Function-Risk-Benefit-Analysis-Report-12.14.2015.pdf Accessed 71, 2017

[19] Tversky A, Kahneman D Judgment under uncertainty: heuristics and biases. Science 1974;185(4157):1124-1131 17835457 10.1126/science.185.4157.1124

[20] Research Excellence Framework 2014. Assessment framework and guidance on submissions. 2011

[21] Evans NG, Lipsitch M, Levinson M The ethics of biosafety considerations in gain-of-function research resulting in the creation of potential pandemic pathogens. J Med Ethics 2015;41(11):901-908 26320212 10.1136/medethics-2014-102619 PMC4623968

[22] Wilson DE, Chosewood LC Biosafety in Microbiological and Biomedical Laboratories. 5th ed. Washington, DC: US Department of Health and Human Services; 2009 https://www.cdc.gov/biosafety/publications/bmbl5/bmbl.pdf Accessed 71, 2017

[23] US Department of Health and Human Services. A framework for guiding US DHHS funding decisions about research proposals with the potential for generating highly pathogenic avian influenza H5N1 viruses that are transmissible among mammals by respiratory droplets. 2013 https://www.phe.gov/s3/dualuse/Documents/funding-hpai-h5n1.pdf Accessed 71, 2017

[24] United States Government Policy for Institutional Oversight of Life Sciences Dual Use Research of Concern. 2014 https://www.phe.gov/s3/dualuse/Documents/oversight-durc.pdf Accessed 71, 2017

[25] Biotechnology and Biological Sciences Research Council, Medical Research Council, Wellcome Trust. BBSRC, MRC and Wellcome Trust position statement on dual use research of concern and research misuse. 2015 https://wellcome.ac.uk/sites/default/files/wtp059491.pdf Accessed 71, 2017

[26] Palmer MJ, Fukuyama F, Relman DA Science governance: a more systematic approach to biological risk. Science 2015;350(6267):1471-1473 26680180 10.1126/science.aad8849

[27] Smith M, Revill J Dual-use biology: how to balance open science with security. Wilton Park Conference Report. WP1260. 2013 https://www.wiltonpark.org.uk/wp-content/uploads/WP1260-Report.pdf Accessed 73, 2017

[28] Patrone D, Resnik D, Chin L Biosecurity and the review and publication of dual-use research of concern. Biosecur Bioterror 2012;10(3):290-298 22871221 10.1089/bsp.2012.0011 PMC3440065

[29] Council of the European Union. Council Regulation No 428/2009 of 5 May 2009 setting up a community regime for the control of exports, transfer, brokering and transit of dual-use items. 2009 Official Journal of the European Union. http://eur-lex.europa.eu/LexUriServ/LexUriServ.do?uri=OJ:L:2009:134:0001:0269:en:PDF Accessed 73, 2017

[30] The Australia Group. Guidelines for transfers of sensitive chemical or biological items. Australia Group website. June 2015 http://www.australiagroup.net/en/guidelines.html Accessed 73, 2017

[31] Selgelid MJ Gain-of-function research: ethical analysis. Sci Eng Ethics 2016;22(4):923-964 27502512 10.1007/s11948-016-9810-1 PMC4996883

[32] Lipsitch M, Plotkin JB, Simonsen L, Bloom B Evolution, safety, and highly pathogenic influenza viruses. Science 2012;336(6088):1529-1531 22723411 10.1126/science.1223204 PMC3467308

[33] Anderson I. Foot and Mouth Disease 2007: A Review and Lessons Learned. London: Stationery Office; 2008 https://www.gov.uk/government/uploads/system/uploads/attachment_data/file/250363/0312.pdf Accessed 73, 2017

[34] No prosecution over Pirbright foot and mouth disease. Times 5 29, 2008 http://www.thetimes.co.uk/tto/news/uk/article1920260.ece Accessed 73, 2017

[35] Arrow KJ Political and economic evaluation of social effects and externalities. In: Margolis J, ed. Analysis of Public Output. Cambridge, MA: NBER; 1970

[36] Pendell DL, Marsh TL, Coble KH, Lusk JL, Szmania SC Economic assessment of FMDv releases from the National Bio and Agro Defense Facility. PLoS One 2015;10(6):e0129134 26114546 10.1371/journal.pone.0129134 PMC4482686

[37] Shavell S Liability for harm versus regulation of safety. J Legal Stud 1984;13:357-374

[38] Berkovitz DM Price-Anderson Act: model compensation legislation? The sixty-three million dollar question. Harvard Environ Law Rev 1989;13(1)

[39] Richardson BJ Mandating environmental liability insurance. Duke Environ Law Policy Forum 2001;12:293

[40] Katzman MT Pollution liability insurance and catastrophic environmental risk. J Risk Insur 1988;55(1):75-100

[41] Jost PJ Limited liability and the requirement to purchase insurance. Int Rev Law Econ 1996;16(2):259-276

[42] Rosenthal M How the Price-Anderson Act failed the nuclear industry. Columbia J Environ Law 1990;15(121)

[43] Schwartz GT Reality in the economic analysis of tort law: does tort law really deter? UCLA Law Rev 1994;(42):377-444

[44] Maynard T, Baxter D Synthetic biology: influencing development. Lloyds Bank Emerging Risk Team. 2009

